# Multi-Sensing Inspection System for Thermally Induced Micro-Instability in Metal-Based Selective Laser Melting

**DOI:** 10.3390/s24175859

**Published:** 2024-09-09

**Authors:** Xing Peng, Rongjie Liao, Ziyan Zhu

**Affiliations:** 1 College of Intelligent Science and Technology, National University of Defense Technology, Changsha 410073, China; liaorongjie@nudt.edu.cn (R.L.); zhuziyan@nudt.edu.cn (Z.Z.); 2 Hunan Provincial Key Laboratory of Ultra-Precision Machining Technology, Changsha 410073, China

**Keywords:** multi-sensing inspection, selective laser melting, thermally induced micro-instability, optical design

## Abstract

Additive manufacturing (AM) excels in engineering intricate shapes, pioneering functional components, and lightweight structures. Nevertheless, components fabricated through AM often manifest elevated residual stresses and a myriad of thermally induced micro-instabilities, including cracking, incomplete fusion, crazing, porosity, spheroidization, and inclusions. In response, this study proposed a sophisticated multi-sensing inspection system specifically tailored for the inspection of thermally induced micro-instabilities at the micro–nano scale. Simulation results substantiate that the modulation transfer function (MTF) values for each field of view in both visible and infrared optical channels surpass the benchmark of 0.3, ensuring imaging fidelity conducive to meticulous examination. Furthermore, the innovative system can discern and accurately capture data pertaining to thermally induced micro-instabilities across visible and infrared spectra, seamlessly integrating this information into a backend image processing system within operational parameters of a 380–450 mm distance and a 20–70 °C temperature range. Notably, the system’s design is harmoniously aligned with the requisites of processing and assembly, heralding a significant advancement in bolstering the inspection effect of thermally induced micro-instabilities for the AM component.

## 1. Introduction

Since entering the 21st century, the traditional industry has gradually transformed into a new manufacturing industry with intelligence, informatization, and automation, and additive manufacturing (AM) technology, also known as “3D printing”, which is a technology with high competitiveness, low cost, and a high manufacturing degree, has been substantially developed [1,2]. AM technology and traditional removal via cutting (i.e., subtractive manufacturing) are different; its core is the layer-by-layer stacking or adding of materials and the gradual building of three-dimensional objects. The process is automatic, direct, fast, and faithful to the initial design ideas, providing a highly efficient and low-cost means of producing prototype parts and realizing new design ideas. AM technology has the potential to shorten processing time, produce complex customized workpieces, repair mechanical parts, and process various free-form components; thus, it has been widely used in aerospace, the military [3], medical equipment, energy, automotive manufacturing, and other fields [3,4,5,6,7].

However, AM technology still has significant limitations in the production process. A thermally induced micro-instability body is an unstable body induced by the thermal process during the selective laser melting (SLM) forming process with low-density zones, stresses, cracks [8,9], and other damages. The size of the pore-type thermally induced micro-instability body ranges from 5 to 20 µm, the size of the extension-type thermally induced micro-instability body ranges from 50 to 500 µm, and the length and openness of the crack-type thermally induced micro-instability body are generally less than 100 µm. Therefore, the appearance of a thermally induced micro-instability body greatly affects the quality of additively fabricated workpieces and reduces the qualification rate of additively fabricated products. Thus, the inspection of thermally induced micro-instability at the micro–nano scale is particularly important.

In recent years, both domestic and international scholars have conducted increasingly in-depth research on the multi-sensing system of thermally induced micro-instability bodies at the micro–nano scale. The main inspection techniques based on the sensors are high-speed camera, ultrasonic technology, X-ray tomography, infrared thermography, photodiode, and pyrometer. Inspection methods utilizing high-speed cameras can detect changes in melt pool dynamics, such as splash and plume, and provide a large amount of data for understanding the AM process. Zhang et al. [10] proposed an off-axis visual monitoring method utilizing a high-speed camera, which proved to be successful in extracting a range of parameters, such as the splash area, the splash rate, and the intensity of the melt pool. Caprio et al. [11] proposed a system based on the monitoring of melt pool surface oscillations for a penetration depth estimation system. Hooper et al. [12] investigated a method for measuring the temporal and spatial resolution required to measure the melt pool surface temperature using a coaxially mounted high-speed camera.

The application of ultrasound in the inspection of thermally induced micro-instabilities capitalizes on the transmission, reflection, and diffraction attributes of ultrasonic waves. This technique ascertains the presence or absence of defects or discontinuities within a component by meticulously capturing parameters such as the propagation waveform, echo characteristics, velocity of the sound, attenuation, and alterations in the spectral attributes of the ultrasonic waveforms as they traverse the component under scrutiny [13]. Hans et al. [14] investigated the use of ultrasonics for online measurements to gain a more accurate understanding of the AM process to understand more accurately the complex dynamics of the manufacturing process and to observe the surface dynamics during the cladding process, and these signals play an important role in the qualitative evaluation of residual stresses. Li et al. [15] investigated the use of ultrasonic arrays for the quality inspection of TC18 titanium alloy parts. X-ray computed tomography (XCT) is a non-destructive volumetric imaging technique that measures the external and internal morphology of a part to obtain three-dimensional information with a spatial resolution of a few micrometers [16]. Cai et al. [17] investigated the XCT technique used to characterize the internal structure of a workpiece to enhance the process parameter of material porosity. Lei et al. [18] and others demonstrated that quantitative structural information such as powder injection, phase transition, melt pool shape or size, and powder solidification can be obtained using XCT imaging and diffraction techniques.

Thermally induced micro-instability body inspection using near infrared (NIR) cameras is undertaken because the rapid solid–liquid–solid transformations during the AM process result in large temperature gradients, which tend to cause defects such as cracks and porosity in metal parts, which seriously affects the component quality. Ye et al. [19] utilized a NIR camera to study the plume and splash feature changes with changes in laser power and scanning speed and verified the feasibility of the monitoring system. A photodiode converts optical radiation into a voltage signal, which can effectively measure the melt pool behavior during the AM process. Any change in the input thermal parameters during the AM process results in a direct change in the thermal radiation. Therefore, photodiodes and pyrometers are utilized to measure radiation. Bisht et al. [20] first proposed a new tool for melt pool monitoring for the SLM process.

However, these conventional inspection methodologies predominantly rely on the isolated utilization of optical, acoustic, thermal, electrical, and other sensory signals. This approach is hampered by subdued signal intensity, an insufficiency of informational richness, and inherent limitations, thereby posing challenges in effectively inspecting the thermally induced micro-instability bodies of workpieces under complex operational conditions. Heat transfer is the driving force behind the laser AM process, with the formation and dynamic behavior of the melt pool, the cooling and solidification of the liquid metal, and the thermal cycling of the solidified layers all related to heat transfer. The complex temperature history during laser AM directly affects the microstructure, residual stresses, and deformation of the components. A uniform temperature distribution contributes to the formation of high-quality components, while an uneven temperature distribution can compromise the structural integrity and quality of the components. Therefore, studying thermal behavior using infrared imaging inspection is of great significance for ensuring the quality of laser AM components. Infrared imaging possesses excellent penetration ability and thermal contrast, being less affected by complex conditions such as powder splashes. However, it struggles to image the detailed information of thermally induced micro-instabilities, resulting in lower inspection precision. In contrast, high-resolution visible light imaging can provide rich detail, facilitating the inspection and feature extraction of optical signals produced by powder layers, splashes, solidified layers, and thermally induced micro-instabilities such as pores and cracks, ensuring the system’s inspection effectiveness. Nevertheless, its imaging quality is easily disturbed by complex environments, making it difficult to detect thermally induced micro-instabilities obscured by powder or overwhelmed by high reflection light. Therefore, this study proposes a cutting-edge multi-sensing inspection system meticulously engineered for the fusion of micro–nano-scale visible and infrared imaging, aimed at detecting thermally induced micro-instabilities in components produced through AM. By employing the multimodal inspection of various signals, it is possible to characterize the fundamental characteristic parameters of thermally induced micro-instabilities during the laser AM process comprehensively. This effectively overcomes the challenges of strong interference from complex conditions such as high temperatures and powder splashes during the laser AM process.

This paper is organized as follows: Section 2 presents the design concept and specifications of the multi-sensing inspection system; Section 3 illustrates the optical design and evaluation of the multi-sensing inspection system; Section 4 and Section 5 show the thermal analysis and tolerance analysis of the multi-sensing inspection system, respectively; and finally, Section 6 concludes this paper.

## 2. Design Concept and Specifications of the Multi-Sensing Inspection System

**Figure 1:**
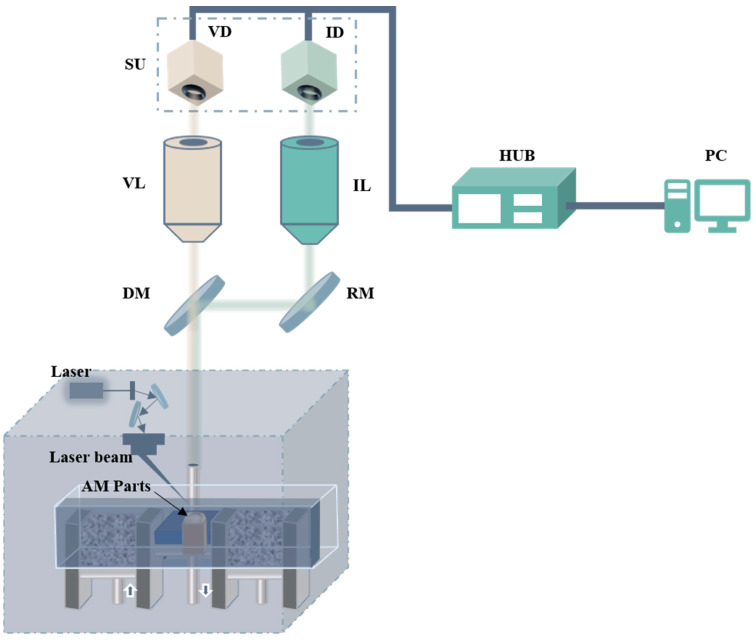
Schematic diagram of the multi-sensor inspection system: IL: infrared imaging channel; VL: visible imaging channel; DM: beam splitter; RM: reflective mirror; ID: infrared imaging detector; VD: visible imaging detector; HUB: hub; PC: computer; SU: sensor unit.

The multi-sensing inspection system mainly contains a visible imaging channel (VL) and an infrared imaging channel (IL), as shown in Figure 1. The visible imaging channel includes a visible light imaging objective set and a CMOS image sensor of Mingkang Optics with a resolution of 6954 × 4822. The infrared imaging channel includes an infrared imaging objective set and an InGaAs image sensor of Mingkang Optics with a resolution of 350 × 256. Also included are a beam splitter with a 30 mm radius and a reflector, as well as a hub and PC image-processing computer. The design parameters of visible and infrared optical imaging channels are illustrated in Table 1.

The working waveband of the visible channel is 0.49–0.51 μm, and the working waveband of the infrared channel is 0.9–1.7 μm. Upon the multi-sensing inspection system’s acquisition of workpiece information, surface feature data undergo refraction via beamsplitter DM and reflection via reflector RM. Subsequently, 50% of the transmitted light is directed into the visible imaging channel through DM, where it is captured by detector VD. The remaining transmitted light is reflected by reflector RM into the infrared imaging channel, with its 50% transmission being collected by detector ID. This image data are then consolidated through the HUB and transmitted to a computer for subsequent machine vision recognition and processing.

In the parameter design phase, the initial step involves ascertaining the maximum resolving power and inspection range necessary for the system’s normal operation. This determination guides the selection of imaging chips. Subsequently, by synthesizing commonly utilized metrics from actual design scenarios, the focal length and aperture number of the system are established. Employing these foundational data, pertinent equations are then used to calculate additional optical system indicators while ensuring that the design parameters fulfill the imaging prerequisites of the optical system. The choice of focal length and object resolution bears a significant correlation with the field of view angle size. The focal length’s magnitude is contingent upon the inspection area’s dimensions, operational distance, and image sensor size.

### 2.1. Optical Parameters of the Visible Imaging Channel

The optical system’s maximum resolution can be defined by the smallest attainable Airy disk size. Furthermore, the system’s maximum resolution ought to be 10^−6^ m or finer, surpassing the imaging chip’s minimal computed pixel size. Consequently, the imaging chip’s pixel size must not exceed 10 μm. To address the design criteria delineated in this chapter, a CMOS sensor with a 1/1.7″ model was selected for the chip. This sensor encompasses dimensions of approximately 7.4 mm × 5.6 mm, a diagonal length of 11 mm, an area of 42 mm^2^, and a pixel size of 1.1 μm.

According to the concept of the aperture angle of an optical system, the magnitude of the field of view can be calculated via Equation (1) [21]:

(1) $$2\theta =2arctan\left(\frac{y}{f}\right),$$

where *θ* is the half field of view angle, *y* is the half image height of the system, and *f* is the focal length of the system. The field of view angle in the horizontal and vertical direction of the visible imaging channel is calculated to be 8.46°, and 6.42°, respectively.

The object-side field of view area can be calculated based on the focal length, working distance, and image sensor size:

(2) $$2Y=2\frac{y\cdot D}{f},$$

where *Y* is the half-width of the field of view region, *D* is the working distance, *y* is the half-image height of the system, and *f* is the focal length of the system. The width of the field of view region in the horizontal and vertical direction of the visible imaging channel is calculated to be 59.20 mm and 44.80 mm, respectively.

The maximum resolution of the system can be calculated as

(3) $$\alpha =f\cdot \frac{1.22\lambda \;}{d},$$

where *α* is the minimum Airy disk size, *λ* is the wavelength of the incident ray, *f* is the focal length of the system, and *d* is the diameter of the entrance pupil. *α* can be simplified to 1.22 *λ*F, and *F* is the aperture number. In the present design, the operational band is meticulously engineered to span 0.49 μm to 0.51 μm. To guarantee superior imaging quality and dependable inspection of thermally induced micro-instability bodies, a 2 × 2 pixel array is employed as the fundamental computational image element. Consequently, the smallest Airy disk size must be less than this fundamental computational image element to uphold imaging integrity:

(4) α=1.22·0.51·F ≤2.2.

Thus, *F* ≤ 3.54. According to the illuminance calculation equation for optical systems [22]:

(5) $$E=\frac{\pi }{4}\delta L{\left(\frac{d}{f}\right)}^{2},$$

where *δ* is the transmittance of the optical system, *L* is the luminance of the target surface, *d* is the diameter of the entrance pupil, and *f* is the focal length. 

The magnitude of *F* significantly influences the optical system’s performance. As *F* increases, there is a corresponding increase in the optical system’s aperture, enabling a greater intake of light and consequently larger illumination. However, this enlargement leads to an augment in the lateral size of the optical components, which inevitably results in increased costs for both the design and installation of the optical system [23]. After comprehensive consideration, the aperture number F2.8 is selected for the design.

The maximum resolution *α* = 1.74 ≤ 2.2 is calculated via Equation (4). It can then meet the requirements of illumination and resolving power. Concurrently, to guarantee superior imaging quality, upon the completion of optical system optimization, it is imperative that the relative illumination exceeds 95%. Furthermore, the object image aberration must be curtailed to below 0.4%, ensuring precise and consistent imaging results.

### 2.2. Optical Parameters of the Infrared Imaging Channel

The focal length of the infrared channel is selected to be consistent with that of the visible channel, and a 50 mm focal length is selected. To satisfy the above conditions, an InGaAs infrared image sensor with a larger inspection wavelength is selected; its pixel number is 350 × 256, the size is 10.50 mm × 7.68 mm, and the minimum calculated pixel size is 30 μm. According to Equation (1), the field of view angle in the horizontal and vertical direction of the visible imaging channel is calculated to be 11.98° and 8.78°, respectively.

According to Equation (2), the width of the field of view region in the horizontal and vertical direction of the visible imaging channel is calculated to be 84.00 mm and 61.44 mm, respectively.

From the parameters of Table 1, the wavelength band of the infrared imaging channel is 0.9–1.7 μm, and the center wavelength can be set to 1.3 μm, which can be calculated as the minimum Airy disk size of the infrared imaging channel, which can be calculated by Equation (3). Thus, F ≤ 18.92. To guarantee sufficient luminosity and ensure the infrared channel possesses a substantial relative aperture, the selection of an aperture number *F* is optimized for maximum feasibility. Consequently, an *F* value of 15 is chosen to fulfill these criteria. The field of view on the object side, as determined by the aforementioned parameters, measures 84.00 mm × 61.44 mm. The maximum resolution, α = 23.79, is less than or equal to 30, thus fulfilling the requisite standards for illumination and resolving power.

## 3. Optical Design and Evaluation of the Multi-Sensing Inspection System

### 3.1. Optical Design and Performance Evaluation of Visible Imaging Channel

In the design of the visible imaging channel, a symmetrical optical configuration is preferred, taking into account the demands for high inspection accuracy, a compact focal length ratio, a streamlined structure, and a large relative aperture. The conventional concentric spherical lens structure offers inherent advantages; as concentric spherical lenses lack an optical axis, concerns regarding field-related aberrations are negated. This simplification allows for focus solely on the correction of longitudinal chromatic aberration, spherical aberration, and spherical chromatic aberration, thereby reducing the secondary optical system’s complexity [24]. However, the utilization of concentric spherical lenses presents three significant practical challenges. First, the structural specifications of the lens, with a maximum thickness of approximately 100 mm and a maximum aperture of roughly 236 mm, greatly exceed those of standard glass blanks, which are typically 50 mm in thickness and rectangular in shape. Consequently, glass manufacturers are required to invest over 100,000 yuan and dedicate around three months to produce these specialized optical glass blanks. Second, the large aperture inherent to the concentric spherical lens structure necessitates the assembly of four mirror groups, demanding an exceptionally minute lens processing accuracy of only 0.01 mm. This precision level substantially elevates the complexity of lens fabrication. Third, the precise alignment required for gluing the four lenses into a coaxial concentric structure poses further challenges. The permissible eccentricity is approximately 0.5, a margin so narrow that it complicates loading and adjustment significantly. Even a slight surge in eccentricity exponentially diminishes the final imaging quality, making the task of maintaining coaxial concentricity exponentially more difficult [25].

The double Gaussian structure optical system is a typical symmetric telecentric optical structure, characterized by a diaphragm in the middle of the system and lens groups on both sides of the diaphragm that are symmetric or approximately symmetric about the diaphragm. There is almost no vertical aberration, which enables efficient and optimal correction of aberrations, coma, and magnification chromatic aberration.

**Figure 2:**
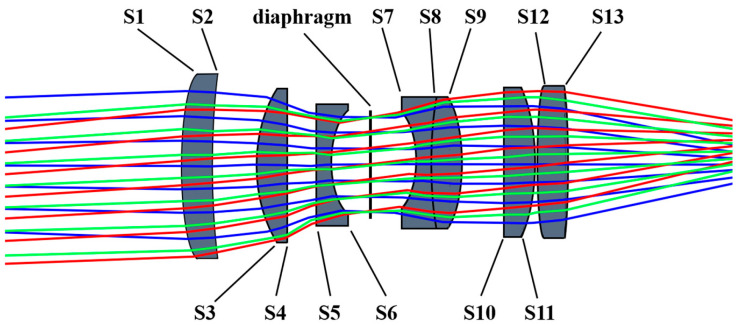
Optical path diagram of the visible imaging channel.

ZEMAX OpticStudio is employed to input parameters that correspond to the evaluation function and optimization variables in the multi-sensing inspection system design. Subsequently, the system refines the lens thickness, surface parameters, and materials through iterative looping with weighted corrections. The field curvature, chromatic aberration, spherical aberration, and dispersion parameters of the system are calibrated to meet the designed parameters to ensure the capability of thermally induced micro-instability body inspection. After the above optimization design, the optical path diagram of the visible imaging channel is shown in Figure 2 and the parameters of each object surface of the visible imaging channel are shown in Table 2.

In general, a quantitative description of the quality of an optical system can be made by analyzing modulation transfer function (MTF) curves, field curvature/distortion, spot diagram, enclosed energy, relative illuminance, and wavefront graphs. The MTF curve of an optical system is a quantitative description of the resolving power of the lens or rather the clarity of the lens image (including both resolution and sharpness). MTF is a lens characteristic function that must be considered in the design of an optical system, and the optical transfer function reflects the imaging quality of the optical system in a comprehensive manner [26]. $MTF=\left(I_{max}-I_{min}\right)/\left(I_{max}+I_{min}\right)$, *I_max_* and *I_min_* denote the maximum and minimum brightness, respectively.

**Figure 3:**
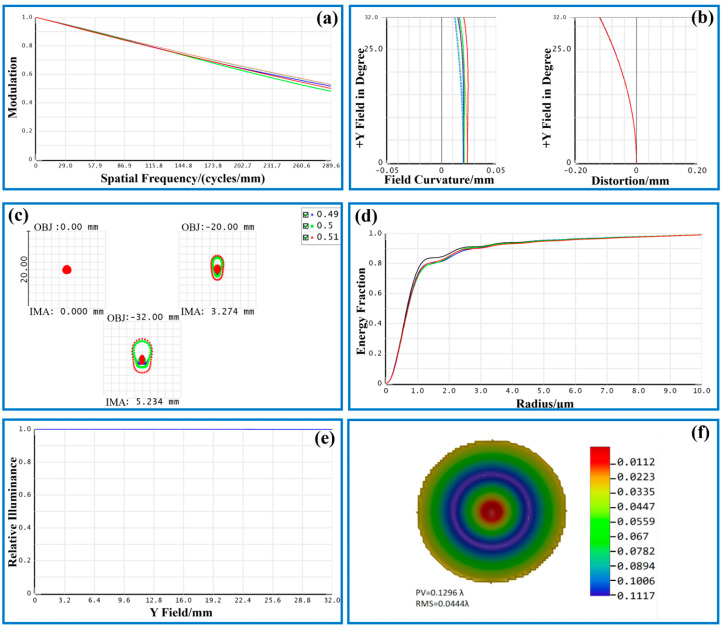
The imaging quality evaluation of the visible channel: (**a**) MTF diagram; (**b**) field curvature and distortion diagram; (**c**) spot diagrams; (**d**) enclosed energy diagram; (**e**) relative illuminance diagram; (**f**) wavefront map.

According to the Nyquist sampling theorem, the cutoff frequency of the image sensor to detect the target is

(6) $$NL=\frac{1000}{5\sigma },$$

where *σ* is the image sensor image element size. The cutoff frequency of the visible imaging is calculated to be 227.27 lp/mm. Figure 3 shows the image quality evaluation results of the visible channel imaging system. As depicted in Figure 3a, at the cutoff frequency of 227.27 lp/mm, the MTF values for the visible imaging channel in the on-axis field of view, 0.6 field of view, and 1.0 field of view all exceed 0.3. Consequently, the design outcomes of the visible imaging channel are satisfactory, and the imaging quality fulfills the prescribed design criteria.

The field curvature/distortion map in ZEMAX is ascertained by tracing the Z-coordinate of the near-axis image plane through meridional ray tracing in the X and Y directions (sagittal and tangential planes, respectively). This involves measuring the distance between the near-axis focal plane and the system image plane’s Z-coordinate. As depicted in Figure 3b, the field curvature and distortion diagram for the visible imaging channel are presented, where the left side illustrates the field curvature. The spacing between the T and S curves of the connected wavelengths denotes the magnitude of dispersion, while the horizontal axis represents the field curvature value. The maximum field value of the system is only 0.02, and the gap between the field curvature value and the dispersion of each working wavelength is relatively small, which would not affect the clarity and quality of the picture received by the sensor. The maximum distortion of the system is only −0.12%, which achieves the design requirements.

Spot diagrams, as a relatively prevalent evaluation method, have been extensively employed in the assessment of contemporary optical systems. When numerous light rays from a single point traverse an optical system, aberrations prevent these rays from converging at an identical location on the image plane. Consequently, this results in a dispersion pattern distributed across a specific area, which is referred to as the spot diagram. As illustrated in Figure 3c, the spot diagram for the visible imaging channel is presented. The various colors on the diagram denote different operational wavelengths: 0.49 μm, 0.5 μm, and 0.51 μm, respectively. The depicted fields of view correspond to the on-axis field of view, the 0.6 field of view, and the 1.0 field of view of the visible imaging channel, respectively. Each spot is smaller than the Airy disk size. The peripheral region of the field of view exhibits a higher degree of light dispersion. However, the majority of the energy is concentrated within the Airy disk. The system’s imaging clarity fulfills the design criteria and meets the specified requirements.

The enclosed energy analysis provides an accurate representation of the light energy distribution on the imaging surface. This analysis measures the enclosed circle energy as a function of the distance from the center of gravity of the image’s primary light or object point. It expresses this energy as a percentage of the total energy, offering a comprehensive depiction of the system’s energy dispersion. A higher concentration of energy density acceptance within the system corresponds to improved signal feedback in the optical imaging system [27]. Figure 3d illustrates that over 80% of the system’s energy is focused within the smallest image element size of 2.2 μm. Consequently, the energy concentration in the visible imaging channel is sufficiently high to fulfill the demands of thermally induced micro-instability inspection.

Relative illuminance is the ratio of illuminance at different coordinate points on the image sensor image plane to the center coordinate point, and this ratio is strongly affected by aberration, vignetting, and pupil aberration [28]. If the relative illuminance of the optical system is smaller, it is more likely to produce overexposure or underexposure problems, which seriously affect the image processing in visual inspection and the feature extraction of the inspection target. Figure 3e shows the relative illuminance curve of this optical system, and the relative illuminance is greater than 99.00%, which meets the design requirements.

The wavefront map allows for direct reading of the peak-to-valley (PV) value of the current wavefront aberration through the displayed data. Theoretically, a PV value less than λ/4 indicates that the optical imaging system has good optical quality [17]. By default, the wavefront pattern is displayed in pseudo-color, and it is orthogonal to the rays. As observed in Figure 3f, the peak-to-valley (PV) value is 0.1296 λ, and its root mean square (RMS) value is 0.0444 λ. These values fulfill the design requirements. To enhance the reliability of the multi-sensing thermally induced micro-instability body inspection system, multiple structures are used to optimize the imaging performance of the visible imaging channel at different working distances, and the design of the optical structure for reliable imaging in the range of 380–450 mm working distance.

**Figure 4:**
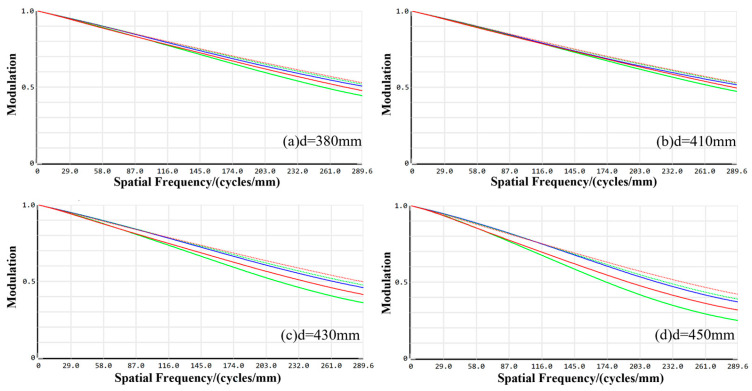
The MTF profiles of the visible imaging channel at different working distances.

As shown in Figure 4, the MTF of the visible channel optical imaging system under each field of view is relatively uniform, and the MTF values of the visible channel imaging system under the cutoff frequency in the on-axis field of view, 0.6 field of view, and 1.0 field of view at a working distance of 380 mm are 0.5454, 0.5654, 0.5956, and 0.6356, all of which are much greater than 0.3. The MTF values are 0.5786, 0.5976, 0.6045, 0.0356, and 0.6356 under the cutoff frequency at a working distance of 410 mm. The MTF values of the visible imaging channel under the cutoff frequency in the on-axis field of view, 0.6 fields of view, and 1.0 field of view are 0.5786, 0.5976, 0.6045, and 0.6654, and all are much larger than 0.3. When the working distance is 430 mm, the MTF values under the cutoff frequency in the on-axis field of view, 0.6 field of view, and 1.0 field of view are 0.4564. The MTF values of the visible imaging channel at the cutoff frequency in the on-axis field of view, 0.6 field of view, and 1.0 field of view are 0.3511, 0.3912, 0.4190, and 0.5572, which are all greater than 0.3. The MTF values of the entire visible channel at the spatial frequency are all greater than 0.3 at the working distance of 450 mm. The MTF values at the spatial frequency of the entire visible channel are all greater than 0.3.

**Figure 5:**
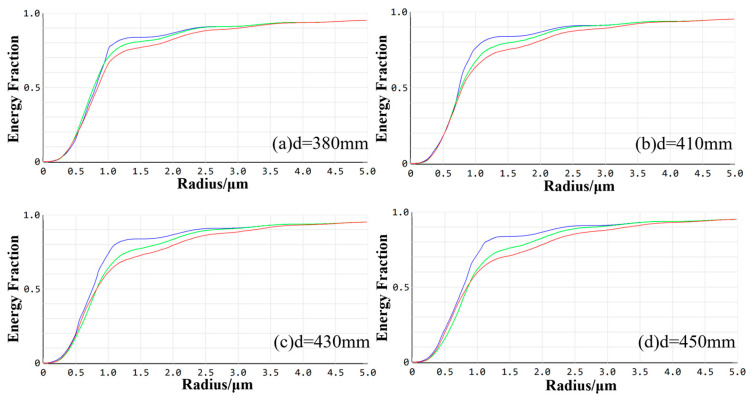
The enclosed energy of the visible imaging channel at different working distances.

Figure 5 illustrates the enclosed energy for the visible imaging channel. At working distances of 380 mm, 410 mm, 430 mm, and 450 mm, the optical energy encapsulated within the smallest calculated image element of 2.2 μm is 87.44%, 82.89%, 81.07%, and 80.16%, respectively, for each field of view. All values exceeded 80%, indicating that the imaging quality of the optical system conforms to the design requirements at these specified working distances.

### 3.2. Optical Design and Performance Evaluation of Infrared Imaging Channel

The field curvature, chromatic aberration, spherical aberration, and dispersion of the system are calibrated to meet the designed parameters to ensure the capability of thermally induced micro-instability body inspection. In the design of the infrared imaging channel system, considering the small focal length ratio, compact structure, and large relative aperture of the system, the same symmetrical optical structure consistent with the description of the visible channel is chosen for the design.

**Figure 6:**
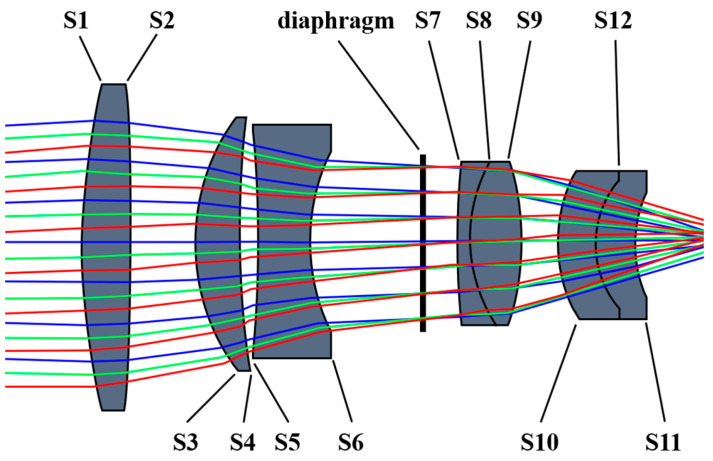
Optical path diagram of the infrared imaging channel.

Following the optimization design outlined above, the optical path diagram for the infrared imaging channel is presented in Figure 6, and the parameters of each object surface in the infrared imaging channel are listed in Table 3. To ascertain whether the system’s imaging performance fulfills the design specifications, further evaluations are required. These assessments include the modulation transfer function, enclosed energy diffraction, field curvature and distortion diagrams, and spot diagrams.

**Figure 7:**
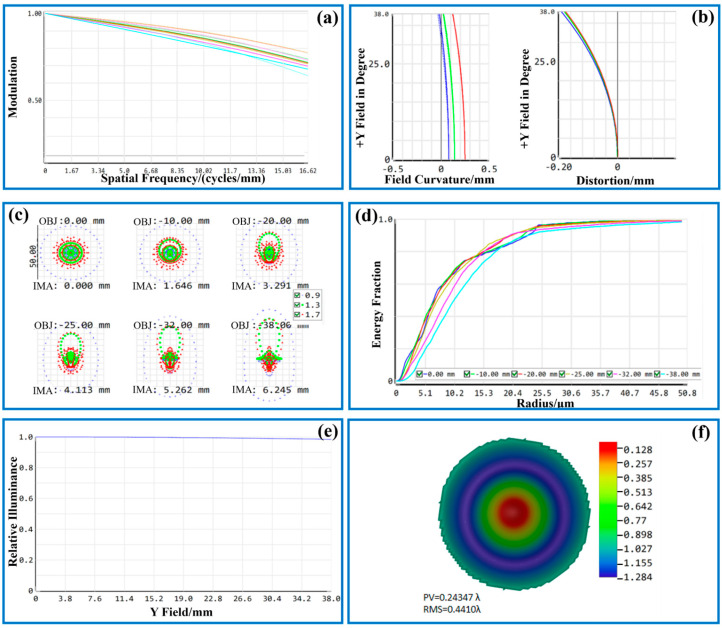
The imaging quality evaluation of the infrared channel: (**a**) MTF diagram; (**b**) field curvature and distortion diagram; (**c**) spot diagrams; (**d**) enclosed energy diagram; (**e**) relative illuminance diagram; (**f**) wavefront map.

As shown in Figure 7, the MTF curve, field distortion, spot diagrams, energy enclosed energy diffraction, relative illumination, and wavefront graphs of the infrared imaging system are shown at a working distance of 400 mm and an ambient temperature of 25 °C.

As shown in Figure 7a, the MTF curves at the cutoff frequency for each preset field of view have a value greater than 0.5; thus, the imaging quality of the infrared channel meets the design requirements. As illustrated in Figure 7b, the field curvature/aberration diagram of the infrared imaging channel reveals that the maximum field curvature value of the system is 0.26. This value is insufficient to impact the clarity of the image captured by the infrared channel sensor. Furthermore, the maximum distortion of −0.18% is significantly below the design threshold. The extent of image distortion is negligible and does not compromise machine vision recognition, thereby satisfying the design requirements.

Figure 7c displays the spot diagrams for the infrared imaging channel. Given that the minimum pixel size of the sensor is 30 μm, the variations in the spot diagram values across different fields of view are marginal. The preset RMS radius for each field of view is 19.603 μm, 19.693 μm, 20.297 μm, 21.046 μm, 22.869 μm, and 25.292 μm, all of which are smaller than the image element size of 30 μm, which demonstrated that the designed structure can be effectively worked to meet the design requirements. As presented in Figure 7d, more than 90% of the energy of the infrared imaging channel is concentrated in the pixel size of 30 μm or less, the energy is more concentrated to meet the imaging requirements. As shown in Figure 7e, the relative illumination curve is greater than 98%, reaching 96% of the design requirements, and the image sensor can be obtained on the image with uniform illumination. Figure 7f shows the wavefront diagram of the infrared imaging channel system, and the PV value of 0.2434 λ is less than the 1/4 λ RMS value of 0.4410 λ, which indicates that the optical imaging system has a good optical quality and meets the design requirements.

Upon integrating the aforementioned icons and data analysis, it is evident that the infrared imaging channel exhibits exceptional imaging quality and superior optical performance. This not only meets the design requirements but also fulfills the demands of machine vision for image inspection acquisition of thermally induced micro-instability.

**Figure 8:**
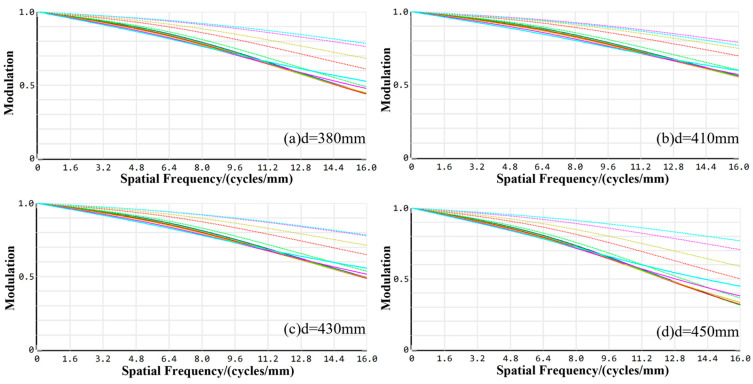
The MTF profiles of the infrared imaging channel at different working distances.

Similar to the optimization process of the visible imaging channel, multiple structures are employed to enhance the imaging performance of the infrared imaging channel across different working distances. This ensures a robust optical structure design for consistent imaging within the operational range of 380 mm to 450 mm. Figure 8 presents the MTF curves for the infrared imaging channel at various working distances.

The values of MTF at the cutoff frequency at a working distance of 380 mm are 0.4451, 0.4643, 0.5109, 0.6348, and 0.6543 for the on-axis field of view and 0.3, 0.5, 0.7, and 1.0 fields of view, respectively. At the working distance of 410 mm, the cutoff frequency values are 0.6643, 0.6690, 0.7532, 0.7650, and 0.8003 for on-axis field of view, and the 0.3, 0.5, 0.7, and 1.0 field of view values are 0.6643, 0.6690, 0.7532, 0.7650, 0.8003, respectively. At the working distance of 430 mm, the MTF values at the cutoff frequency in all fields of view are all larger than 0.3. The MTF values at the cutoff frequency of 450 mm are 0.3926, 0.3977, 0.4593, 0.4903, 0.5891 for the on-axis field of view and 0.3, 0.5, 0.7, and 1.0 fields of view, respectively.

**Figure 9:**
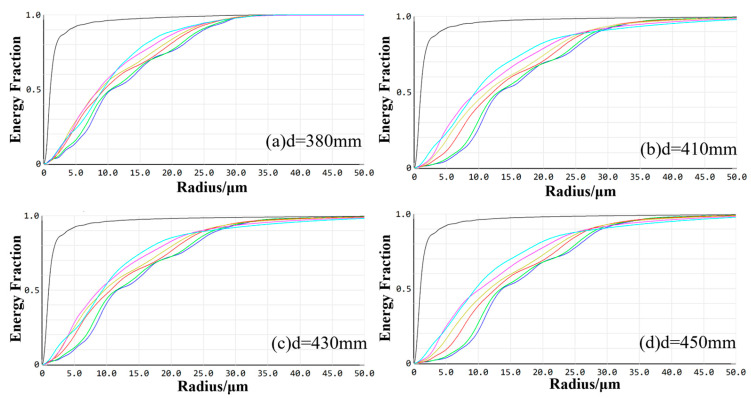
The enclosed energy of the infrared imaging channel at different working distances.

Figure 9 illustrates the enclosed energy for the infrared imaging channel. The optical energy encapsulated within a 30 μm image element under each field of view at operating distances of 380 mm, 410 mm, 430 mm, and 450 mm is 97.88%, 93.84%, 92.33%, and 90.50%, respectively. All these values exceed 80%, indicating that the system maintains high imaging quality across a broad range of working distances. Consequently, the imaging performance of the infrared optical system satisfies the design requirements.

## 4. Thermal Analysis of the Multi-Sensing Inspection System

### 4.1. Thermal Analysis of the Visible Imaging Channel

**Figure 10:**
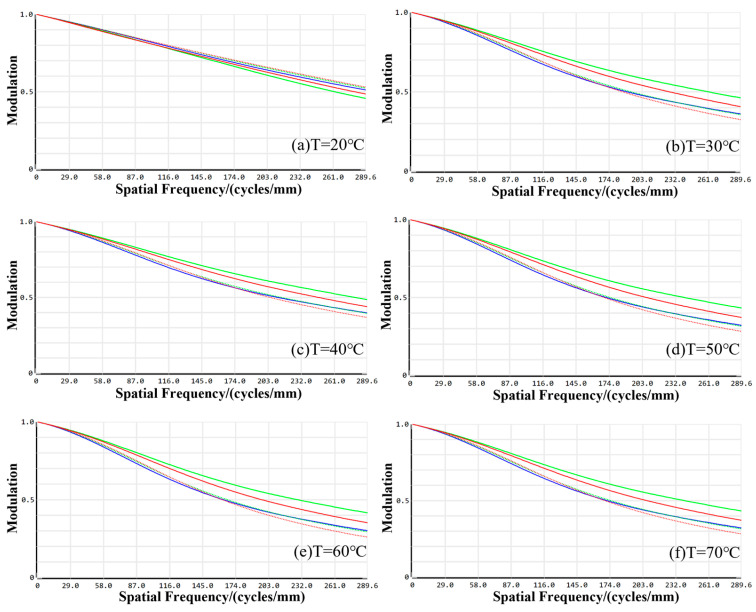
MTF profiles of the visible channel imaging system at various working temperatures.

Considering the application of the designed visible imaging channel in laser AM visual inspection, which involves fluctuations in the working environment temperature, a thermal analysis was conducted on the optical system at room temperature and elevated working temperatures. To ensure optimal imaging performance, the lens structure of the imaging system was meticulously optimized and analyzed using passive thermal aberration design across six distinct configurations. These configurations corresponded to the following working environment temperatures: T = 20 °C; T = 30 °C; T = 40 °C; T = 50 °C; T = 60 °C; and T = 70 °C. Figure 10 shows the MTF curves of the visible imaging channel at each working ambient temperature.

During the optimization procedure, consistent parameter optimization weights were maintained across each temperature configuration. At a working temperature of 20 °C, the MTF values for the visible imaging channel in the on-axis field of view, 0.3 field of view, 0.6 field of view, and 1.0 field of view were 0.5532, 0.5644, 0.5865, and 0.6432, respectively. As the working temperature increased to 30 °C, the MTF values altered to 0.4097, 0.4356, 0.5359, and 0.5911. At 40 °C, the values became 0.4434, 0.4932, 0.5024, and 0.5532. Elevating the temperature to 50 °C resulted in MTF values of 0.3978, 0.4253, 0.4923, and 0.5644. Further increases to 60 °C yielded 0.3451, 0.3705, 0.4015, and 0.4922, while at 70 °C, the values were 0.3312, 0.3563, 0.4196, and 0.5574. Notably, the MTF values across all configurations in the full field of view remained above 0.3, indicating commendable imaging quality that satisfies the design prerequisites for the visible imaging channel.

### 4.2. Thermal Analysis of the Infrared Imaging Channel

**Figure 11:**
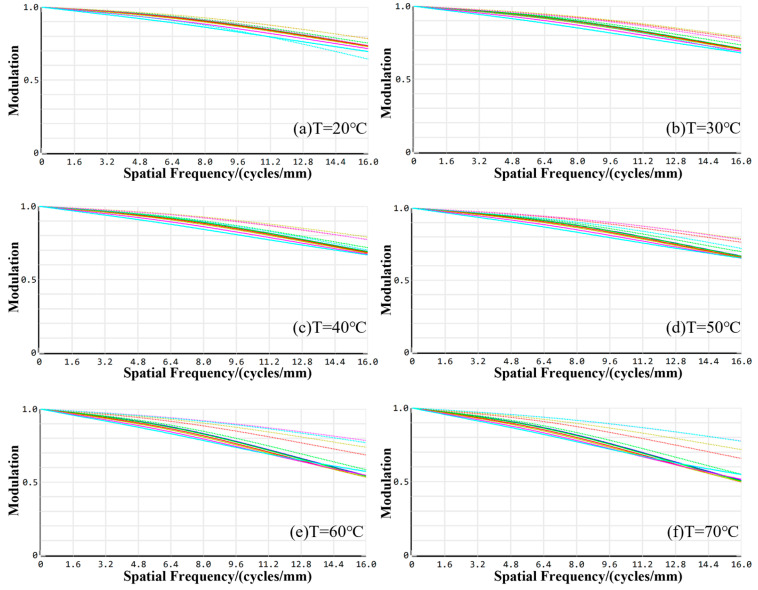
MTF profiles of the infrared channel imaging system at various working temperatures.

The MTF profiles of the infrared imaging channel at various working temperatures are presented in Figure 11. At a working temperature of 20 °C, the minimum MTF value across the on-axis, 0.3, 0.5, 0.707, and 1.0 fields of view is 0.7128, with a maximum difference of 0.0625, indicating favorable dispersion. At 30 °C, the minimum MTF value remains consistent at 0.7503 across all fields of view, with a slight increase in the maximum difference to 0.0636, still demonstrating commendable dispersion. Elevating the temperature to 40 °C results in a minimum MTF value of 0.6826, with a reduced maximum difference of 0.0326, suggesting enhanced dispersion. Further increases to 50 °C and 60 °C yield minimum MTF values of 0.6908 and 0.5873, respectively, with corresponding maximum differences of 0.0414 and 0.0636. Notably, the dispersion of the MTF curve incrementally grows as the temperature rises. At the elevated temperature of 70 °C, the minimum MTF value falls to 0.4391, accompanied by a substantial maximum difference of 0.2711, yet this remains within an acceptable range. Importantly, the MTF values across the full field of view for all temperature settings surpass the threshold of 0.3. The peak MTF difference across diverse fields of view is 0.2711, which is within permissible limits, ensuring effective imaging. As illustrated in Figure 11, the energy encapsulation within the image element size at all operational distances exceeds 80%, confirming that the imaging quality satisfies the stringent requirements of the infrared imaging channel design.

## 5. Tolerance Analysis of the Multi-Sensing Inspection System

In light of the aforementioned optical design optimization results for the visible imaging channel, it becomes imperative to address the errors introduced during the fabrication and assembly phases prior to practical implementation. This necessitates conducting a comprehensive tolerance analysis to ascertain the system’s error margin, ensuring that it aligns with the imaging system’s specifications. Throughout the tolerance analysis of this particular design, initial tolerance values are assigned to the optical system’s optical and structural parameters, supplemented by compensation parameters. Concurrently, a Monte Carlo analysis is performed, utilizing the MTF average as the criterion for analysis and evaluation. Based on the analytical outcomes, the tolerances for specific parameters are either constricted or relaxed as needed, yielding tolerance analysis results that conform to the stringent standards of fabrication and assembly. The tolerance values of the visible imaging channel are shown in Table 4. Among them, the lens radius tolerance is ±0.08 mm, the center thickness tolerance is ±0.08 mm, the surface eccentricity tolerance is ±0.05 mm, the surface tilt tolerance is ±0.05 mm, the surface irregularity tolerance is 0.2 fringe, the element eccentricity tolerance is ±0.05 mm, the element tilt tolerance is ±0.05°, the refractive index tolerance is ±0.001, and the Abbe tolerance is ±1%.

Tolerance values for the infrared imaging channel are also presented in Table 4. The lens radius tolerance is ±0.20 mm, the center thickness tolerance is ±0.20 mm, the surface eccentricity tolerance is ±0.15 mm, the surface tilt tolerance is ±0.15 mm, the surface irregularity tolerance is 0.2 fringe, the element eccentricity tolerance is ±0.3 mm, the element tilt tolerance is ±0.3°, the refractive index tolerance is ±0.001, and the Abbe tolerance is ±1%.

Sensitivity analysis was conducted, complemented by a Monte Carlo simulation executed 2000 times to derive the results. The objective was to constrain the decline in the MTF average value at the cut-off frequency to within a 10% limit. This process identified the top ten tolerance parameters with the most significant impact: TRAD represents the radius of curvature tolerance, TSDY denotes the surface eccentricity tolerance in the Y-direction, TIRX signifies the surface inclination tolerance in the X-direction, TTHI indicates the thickness tolerance, TSDX refers to the surface eccentricity tolerance in the X-direction, TIRY is the surface inclination tolerance in the Y-direction, TEDY stands for the component eccentricity tolerance in the Y-direction, and TEIX represents the component inclination tolerance in the X-direction.

**Figure 12:**
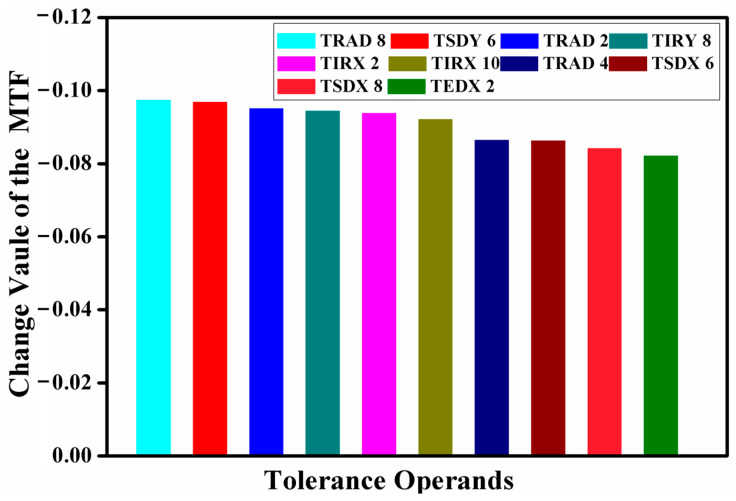
Tolerance analysis of visible channel imaging system.

The tolerance analysis pertaining to the visible imaging channel is presented in Figure 12. According to the analysis, the maximum variation in the MTF value is 9.74%, which is attributed to a radius of curvature tolerance of ±0.08 mm. The declines in MTF values, induced by the ten most significant tolerance items, were found to be 9.74%, 9.69%, 9.51%, 9.44%, 9.38%, 9.21%, 8.65%, 8.63%, 8.42%, and 8.22%, respectively, with a cumulative impact of 0.968. When juxtaposed with the outcomes of the preceding tolerance analysis, it becomes evident that the design of the visible imaging channel not only satisfies the prescribed requirements but also adheres to the stipulated conditions for fabrication and assembly.

**Figure 13:**
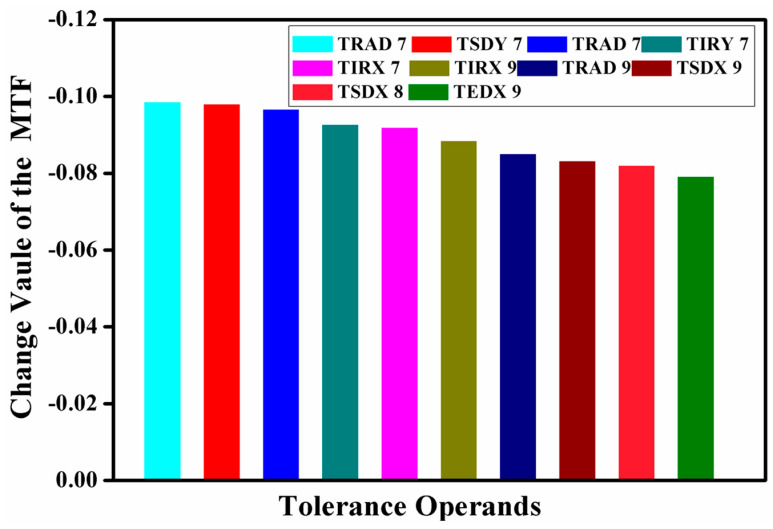
Tolerance analysis results of infrared channel imaging system.

The tolerance analysis pertaining to the infrared imaging channel is delineated in Figure 13. The analytical results reveal that the most substantial variation in the MTF value, measured at 9.84%, arises from a radius tolerance of ±0.20 mm. The decrements in MTF values, precipitated by the ten preeminent tolerance items, are recorded as follows: 9.84%, 9.79%, 9.65%, 9.26%, 9.18%, 8.83%, 8.50%, 8.31%, 8.19%, and 7.90%. When these findings are considered collectively with the preceding tolerance analysis, it becomes evident that the design of the infrared imaging channel not only proscribes the necessary standards but also conforms to the requisite conditions for fabrication and assembly.

## 6. Conclusions

Additive manufacturing (AM) stands unrivaled in its capacity to engineer intricate forms, pioneer functional components, and realize lightweight structures. Yet, components produced via AM frequently exhibit heightened residual stresses and a plethora of thermally induced micro-instabilities, including cracking, incomplete fusion, crazing, porosity, spheroidization, and inclusions. However, traditional methods of thermally induced micro-instabilities inspection in laser AM parts, such as high-speed cameras, infrared thermal imagers, and photodiodes, are only capable of measuring individual sensing signals related to sound, light, and heat. These methods exhibit low signal accuracy, and inadequate informational output. Therefore, this paper proposed a sophisticated multi-sensing inspection system specifically tailored for the inspection of thermally induced micro-instabilities at the micro–nano scale. In accordance with the optical performance prerequisites, the design parameters for the multi-sensing inspection system multi-sensing inspection system, encompassing the visible imaging channel and infrared imaging channel, were meticulously calculated. Subsequently, the optical imaging system was meticulously designed and optimized. To further ensure compliance with processing and assembly requirements, a rigorous tolerance analysis, coupled with a Monte Carlo analysis, was conducted on the optical structures within the multi-sensing inspection system. The simulation results illustrate that the system’s design is harmoniously aligned with the requisites of processing and assembly, heralding a significant advancement in bolstering the inspection effect of thermally induced micro-instabilities for the AM component, which further offers a beneficial and promising solution for the thermally induced micro-instabilities characterization and optimization of processing parameters in laser AM.

## Figures and Tables

**Table 1:** Design parameters of visible and infrared optical imaging channels.

Design Parameters	VL	IL
Wavelength (μm)	0.49–0.51	0.9–1.7
Image sensor type	CMOS	InGaAs
Pixel count	6954 × 4822	350 × 256
Pixel size (μm)	1.1	30
Focal length f (mm)	50	50
F-numbers	2.8	15
Field of view	10.60°	14.80°
Working distance *d* (mm)	400	400
Relative illumination (%)	>95.00	>95.00
Distortion (%)	<0.4	<0.4

**Table 2:** Parameters of optical surfaces of the visible imaging channel.

Surface	Radius of Curvature/mm	Clear Semi-Diameter/mm	Mechanical Semi-Diameter/mm
S1	55.32	16.84	16.84
S2	184.55	16.22	16.84
S3	28.38	14.08	14.08
S4	1216.35	13.44	14.08
S5	−249.61	10.65	10.65
S6	17.58	8.97	10.65
S7	−18.09	9.34	11.96
S8	60.77	11.36	11.96
S9	−27.16	11.96	11.96
S10	−240.52	13.29	13.59
S11	−53.44	13.59	13.59
S12	105.53	13.52	13.52
S13	−211.50	13.20	13.52

**Table 3:** Parameters of optical surfaces of the infrared imaging channel.

Surface	Radius of Curvature/mm	Clear Semi-Diameter/mm	Mechanical Semi-Diameter/mm
S1	120.72	29.45	29.45
S2	−528.18	28.89	29.45
S3	43.35	23.42	23.42
S4	275.97	22.46	23.42
S5	−399.63	21.58	21.58
S6	36.27	16.96	21.58
S7	133.37	14.43	14.64
S8	26.13	14.52	14.64
S9	−45.73	14.64	14.64
S10	29.31	13.29	13.29
S11	18.37	11.26	13.29
S12	24.79	9.72	13.29

**Table 4:** Tolerance values for visible and infrared channel imaging systems.

Tolerance	VL	IL
Radius (mm)	±0.08	±0.20
Thicknesses (mm)	±0.08	±0.20
Surface eccentricities (mm)	±0.05	±0.15
Surface tilt (mm)	±0.05	±0.15
Surface irregularity (fringe)	0.2	0.2
Element eccentricity (mm)	±0.05	±0.3
Element tilt (°)	±0.05	±0.3
Refractive index	±0.001	±0.001
Abbe (%)	±1	±1

## Data Availability

The original contributions presented in the study are included in the article, further inquiries can be directed to the corresponding author.
